# Perceived barriers and facilitators to participation in physical activity for children with disability: a qualitative study

**DOI:** 10.1186/s12887-016-0544-7

**Published:** 2016-01-19

**Authors:** Nora Shields, Anneliese Synnot

**Affiliations:** Department of Rehabilitation, Nutrition and Sport, School of Allied Health, La Trobe University, Melbourne, VIC 3086 Australia; Northern Health, 185 Cooper St., Epping, VIC 3076 Australia; Centre for Health Communication and Participation, School of Psychology and Public Health, La Trobe University, Melbourne, VIC 3086 Australia; Australian & New Zealand Intensive Care Research Centre, School of Public Health and Preventive Medicine, Monash University, Melbourne, VIC 3004 Australia

**Keywords:** Children with disability, Physical activity, Barriers, Facilitators, Qualitative

## Abstract

**Background:**

Children with disability engage in less physical activity compared to their typically developing peers. Our aim was to explore the barriers and facilitators to participation in physical activity for this group.

**Methods:**

Ten focus groups, involving 63 participants (23 children with disability, 20 parents of children with disability and 20 sport and recreation staff), were held to explore factors perceived as barriers and facilitators to participation in physical activity by children with disability. Data were analysed thematically by two researchers.

**Results:**

Four themes were identified: (1) similarities and differences, (2) people make the difference, (3) one size does not fit all, and (4) communication and connections. Key facilitators identified were the need for inclusive pathways that encourage ongoing participation as children grow or as their skills develop, and for better partnerships between key stakeholders from the disability, sport, education and government sectors. Children with disabilities’ need for the early attainment of motor and social skills and the integral role of their families in supporting them were considered to influence their participation in physical activity. Children with disability were thought to face additional barriers to participation compared to children with typical development including a lack of instructor skills and unwillingness to be inclusive, negative societal attitudes towards disability, and a lack of local opportunities.

**Conclusions:**

The perspectives gathered in this study are relevant to the many stakeholders involved in the design and implementation of effective interventions, strategies and policies to promote participation in physical activity for children with disability. We outline ten strategies for facilitating participation.

**Electronic supplementary material:**

The online version of this article (doi:10.1186/s12887-016-0544-7) contains supplementary material, which is available to authorized users.

## Background

Children with disability engage in less physical activity compared to their typically developing peers [1, 2]. Regular participation in physical activity by children, including those with disability, enhances body composition [3], bone health [4, 5], psychological health [6, 7] and promotes social engagement [8]. There are additional therapeutic benefits to participation in regular activity for children with disability [9]. They often have delayed gross motor development, less proficiency in balance and coordination and poor cardiovascular fitness compared to their peers with typical development [10], all of which could potentially be improved by participation in physical activity.

The reasons for lower levels of participation in physical activity among children with disability are complex and multifactorial [11]. The Physical Activity for People with a Disability conceptual model [12] helps to illustrate the relationship between physical activity behaviour, its determinants, and health, including the role of contextual factors (personal and environmental) for people with disability. The model incorporates barriers to and facilitators of physical activity for people with disability by acknowledging multiple determinants of physical activity exist.

The barriers to participation in physical activity have been studied more comprehensively than the facilitators to participation, and include a lack of knowledge and skills, the child’s preferences, fear, parental behaviour, negative attitudes to disability, inadequate facilities, lack of transport, lack of programmes and staff capacity, and cost [13]. Reported facilitators include the child’s desire to be fit and active, skills practice, involvement of peers, family support, close and accessible facilities, opportunities sensitive to the needs to children with disability, skilled staff and information dissemination [13].

The reported barriers and facilitators to participation can differ according to whose views are elicited. Children with disability tend to focus on personal factors, while parents focus on familial, social and policy and programme factors [13]. Most published studies in this area have sought only the perspectives of children with disability or their parents and only a small number have included the views of other stakeholders such as professionals who work in the sport and recreation sector [14, 15]. Personnel from the sports and recreation sector are ideally placed to promote participation in physical activity among children with disability given their role in the design, organisation and delivery of activity opportunities and infrastructure [16–18]. One small study has explored the perspective of sports and recreation industry personnel only [15]. Based on a content analysis of a short survey in a convenience sample of 24 staff, it found the most common perceived barriers were inaccessible facilities, non-inclusive providers, transport, lack of relevant opportunities and cost. The most common perceived facilitators reported were welcoming providers, parental support, inclusive providers, adaptable approaches and accessibility of facilities. The study design was limited, however, as it did not allow for an in-depth analysis of these factors. A richer description of these barriers and facilitators would complement our understanding, and has the potential to identify new ways to enhance participation in physical activity for children with disability.

Therefore, the aim of our study was to explore the barriers and facilitators to participation in physical activity from the perspectives of children with disability, their parents and sports and recreation industry personnel.

## Methods

### Research design

We completed a descriptive study using qualitative methods [19]. The experience of participation in physical activity by children with disability was explored through a series of focus groups with relevant stakeholders (children, parents, and sports and recreation industry personnel). The aim was to draw out their specific experiences of what helped and hindered participation in physical activity for children with disability to inform practice and future research rather than to develop new theory. We chose focus group methods for data collection to take advantage of group interaction to encourage discussion between the participants to compare and contrast their experiences and views.

Ethical approval was obtained from La Trobe University Human Ethics Committee and from the Victoria Government Department of Education and Early Childhood Development. All participants gave written informed consent prior to their participation. Children with disability were invited to provide their own written assent in addition to their parent’s written consent. Participants were assured that their identities would remain anonymous in all reporting of the study and that their personal information would be kept confidential.

### Participants

Purposive sampling was used to select participants representing different participant groups, disability types and geographical locations as there was evidence that these factors might affect the barriers and identified. To be eligible, participants had to be a child with a disability (congenital or acquired) aged 10–18 years, the parent of a child with disability aged between 6–18 years, or a professional working in the sports and recreation sector with people with disability. Children aged 6–9 years were excluded from taking part in the focus groups. It was considered inappropriate to include children 6–9 years in the same group as older children and adolescents (10–18 years) as the younger children may have difficulty contributing at the same level as the older children and adolescents. Participants needed sufficient communication skills (verbal or sign language) to participate in a focus group. Participants were excluded if either they or the person they cared for had an impairment was due to a medical condition (such as epilepsy or cancer) or a learning disability (such as dyslexia).

Participants were recruited through disability groups, sport and recreation groups, therapy services and special schools. These agencies were asked to identify potential participants and forward to them information about the study. Potential participants then contacted the researchers directly. Information about the study was also forwarded by the researchers to people who had participated in a previous study [20] and had indicated their willingness to take part in future studies.

### Focus groups

Three focus groups were held with parents and three with sports and recreation industry personnel at local community venues, schools or therapy centres convenient to each group of participants. An experienced external moderator conducted the focus groups with assistance from one of the researchers. Participants were sent a list of the topics to be discussed one week prior to the focus group, to allow time to reflect and to bring notes if they wished. After group introductions, participants were asked to come up with a list of barriers and facilitators which was transcribed onto butchers paper. As a group, participants were then asked to reflect on and discuss the items in the list. Sessions lasted approximately one hour and were audio recorded (see Additional file 1 for the parent focus group schedule).

Four groups were conducted with children with disability; one group with children with physical disability (cerebral palsy), two groups with children with mild intellectual or developmental disabilities and one group with adolescents with vision impairment. These focus groups were conducted at specialist schools. The sessions commenced with the children introducing themselves and sharing what physical activities they took part in. With the exception of one focus group that included children with vision impairment, a ball was passed between children to encourage everyone to speak, and to discourage more than one child speaking at once. Using their current activities as a reference point, the children were asked to share what made these activities fun and what made them hard. Photographs of children with and without disability taking part in physical activities were also used to help stimulate discussion. These photographs were sourced from Google images and depicted a variety of physical activities including football, swimming, walking, cycling, basketball, horse riding, dancing, sailing, cricket, athletics, gymnastics, and skipping. The children were asked to select an activity depicted in one of the photographs that they had never done before but would like to try, as a way of exploring what some barriers to participation might be.

### Data analysis

Data were transcribed verbatim and examined using thematic analysis [21]. Two researchers independently read the transcriptions several times and coded the data line-by-line to identify emerging concepts. These concepts were derived from the data and were not preconceived. The number of concepts were not restricted and as many concepts were identified as they emerged [22]. These concepts eventually formed the themes that we present in the Results section. NVivo software (Version 8, QSR international) was used to assist coding. Each focus group was analysed separately to ensure the views of that particular focus group were considered. The data of each participant group (i.e. children, parents, sports and recreation industry personnel) was then considered separately to get an overview of the views of that subgroup. After coding, through a consensus process, like concepts were grouped into sub-themes, and these were drawn together to form themes. During these discussions, the researchers took into account if a theme or subtheme represented the views of all participant groups, and rich thick descriptions were used to exemplify sources. When the final list of themes was agreed, the transcripts were re-read and key word searches performed to ensure no relevant aspect of these themes had been overlooked.

### Trustworthiness

Two main strategies were used to enhance the trustworthiness of the data [23]. When the initial data analysis was completed, the list of themes generated was sent to the participants for validation (member checking). Through this process, the participants, including the children with disability, verified the interpretation of the data. Credibility of the data was enhanced by having two researchers independently code and interpret the data, providing a basis for reflective discussions which helped to provide a more complete understanding.

## Results

Sixty-three participants took part in ten focus groups (see Table 1): 23 children with disability (mean age 13.9 ± 1.8 years; 8 girls, 15 boys), 20 parents of children with disability (18 females, 2 males) and 20 professionals who worked in the sports and recreation industry and who worked with children with disability (11 females, 9 males). The children who took part in the focus groups had the following types of disability: cerebral palsy (*n* = 6), vision impairment (*n* = 5), intellectual disability (*n* = 7), developmental delay (*n* = 4), and multiple disabilities (*n* = 1). Four themes emerged from the data (summarised in Table 2).

**Table 1:** Participant characteristics (parent and child focus groups)

Group type	Location	No of participants (male: famle)	Children’s		
			Age (mean ± SD)	Age range (years)	Disability
Children	Metro	4 (3M:1F)	12	12	Cerebral Palsy (*n* = 4)
	Metro	7 (5M:2F)	12.5 ± 0.5	12-13	Intellectual disability (*n* = 3)
					Intellectual disability & Autism Spectrum Disorder (*n* = 2)
					Developmental Delay (*n* = 2)
	Metro	6 (2M:4F)	15.0 ± 1.1	13-16	Developmental Delay (*n* = 2)
					Intellectual disability (*n* = 2)
					Cerebral Palsy & Intellectual Disability (*n* = 1)
					Multiple Disabilities (*n* = 1)
	Mixed	6 (5M:1F)	15.8 ± 1.2	14-17	Vision Impairment (*n* = 4)
					Vision Impairment & Cerebral Palsy (*n* = 1)
					Vision Impairment & Hearing Impairment (*n* = 1)
Parents	Metro	5 (5F)	11.0 ± 2.2	7-12	Cerebral Palsy (*n* = 4)
					Cerebral Palsy & Aphasia (*n* = 1)
	Region^a^	8 (2M:6F)	8.5 ± 3.7	6-17	Intellectual disability (*n* = 3)
					Multiple disabilities (*n* = 3)
					Intellectual disability & Autism Spectrum Disorder (*n* = 2)
					Autism Spectrum Disorder (*n* = 2)
	Metro^a^	7 (7F)	12.3 ± 3.1	7-16	Developmental Delay (*n* = 4)
					Multiple disabilities (*n* = 1)
					Intellectual Disability (*n* = 1)
					Cerebral Palsy (*n* = 1)
					Autism Spectrum Disorder (*n* = 1)

Abbreviations: *Metro* metropolitan, *Region* regional

^a^One or more parents in the group had more than one child with a disability aged between 6 to 18 years

**Table 2:** Emergent themes and subthemes from the semi-structured interviews

Barriers	Facilitators
Theme 1 There are similarities and differences between children with disability and children with typical development	
Longer to develop skills	Positive encouragement from others
Lack of physical skill	One-on-one instruction
Frustration or loss of confidence when child compares self to peers	Children that are motivated to keep fit
	Happy-go-lucky, confident child
It’s harder as children get older	Naturally active child
Need extra support to participate	
Extra costs associated with raising a child with disability	
Theme 2 People make the difference	
Parents lack knowledge or means	Proactive parents
Lack of practical instructor training	Skilled instructors
Negative societal attitudes towards disability	Peer acceptance
	Understand disability
Disability a low priority	Inclusive policies & programs
Parents doubt child’s safety or ability	Family involvement
Parental exhaustion	
Theme 3 One size does not fit all…it’s about choice	
Children and parents are not asked about how they can participate	Inclusive pathways
	Fun & sense of success
Lack of transport	Transport
Distance	Local activities
Lack of activities	Meaningful, appropriate activities
One-off programs	Opportunities at school
Waiting lists	
No quorum	
Theme 4 Communication and connections between stakeholders	
Poor advertising of programs	Word of mouth between parents
Difficulty for program providers finding families	Special schools provide information on activity
Limited partnerships between sectors	Partnerships between schools, activity providers, disability groups and councils

### Similarities and differences

Participants identified a number of barriers to participation that affect children with and without disability. These included children not being interested in physical activity, limited transport, cost and lack of time. Participants described additional barriers that exist for children with disability, such as not being as physically capable as their peers and social barriers such as negative societal attitudes. Parents in particular identified that their child felt a sense of frustration or loss of confidence when they compared their skills with those of their peers with typical development. They described that one-on-one instruction and positive encouragement increased their child’s confidence and skills, and this in turn facilitated on-going participation in physical activity.

*“Now he’s going in the normal class actually, he’s not getting one-on-one attention because he can manage by his own (…) but it took quite a long time.” [Parent #1]*

Participants described that it gets harder for children with disability to participate in physical activity as they get older, as the skill gap widens and sports become more competitive. Parents shared stories of worsening teasing and exclusion by peers as their child aged and of an increasing lack of motivation to take part in physical activity as a result.

*“When he was younger they accepted him very well. But then as he got older and his movements became more awkward (…) so kids pick it up, and kids are cruel” [Parent #2]*

Parents reported that children who were naturally active, loved sports and had a happy go lucky personality were more easily engaged in physical activity. This was corroborated by the children with disability who described their reasons for participating were it was fun, it gave them a sense of success or competence, to keep fit, and to engage in activities with friends, or make new friends.

*“Sport’s my life really, so I play every sport I can have a try at (…) Things are hard for sport but (…) it just doesn’t matter, better to have a crack” [Child #1, aged 12]*

*“Well I play footy because I love getting outside and I love moving around and it’s fun and I always do it with a lot of my mates” [Child #2, aged 16]*

*“It comes down to affordability”* was a key consideration for families and sports and recreation industry personnel. While cost is barrier to participation in physical activity that affects children with and without disability, there was an additional burden for families of a child with disability given the extra expense of caring for a child with disability, a reduced income as parents often worked less and the need for one-on-one attention.

*“I’d put my son in half a dozen activities …., but by the time you pay for speech therapy, occupational therapy (…) there’s not a lot of money leftover” [Parent #3]*

Flexible payment schemes, subsidised programs and access to modified equipment were suggested as ways to facilitate participation.

### People make the difference

The attitudes of people close to children with disability such as families, instructors and peers, were seen as central to their participation in physical activity by all participant groups. Experience of disability was considered to underpin attitudes: when people understood disability they were more likely to be welcoming and supportive of children with disability.

The integral role of families in facilitating their child’s participation in physical activity was highlighted by all participant groups. This included providing financial support, transport, finding suitable activities and encouragement. Participants described how parents needed to be proactive to get their child active; examples included parents being physically involved in the activity, researching available activities, knowing about possible modifications to activities, and advocating for their child.

*“I am involved physically as well as supporting …. I go into the water, I go down to the sand, I run behind the bike to make sure he’s safe” [Parent #4]*

Related to this was the opinion that ‘sporting’ families were better equipped to help children with disability engage in activity as they understood sporting culture and had experienced the benefits of physical activity.

*“My uncle plays as well and my mum and my nana, my uncle is a tennis coach, so I find it easier for me to do” [Child #3, aged 15]*

A reliance on families to facilitate their participation meant that if parents lacked experience in physical activity, or didn’t have the means to get involved, the child would miss out. Parents talked about how tough it can be caring for a child with disability. This was illustrated by comments about their exhaustion and the challenge of finding time to fit physical activity into the family schedule.

*“When you’ve got a child with a disability you’re exhausted all the time, you don’t get a reprieve, we don’t get time off” [Parent #3]*

Some parents expressed doubt about their child’s ability to participate in physical activity, or concern for their child’s safety.

“*I’m scared to put him in the mainstream*” *[Parent #1]*

Other key personnel identified as facilitating participation in physical activity were coaches, instructors, and physical education teachers who were willing and able to modify activities. The view of parents and children, was that success or failure hinged on an instructor’s ability to successfully include a child with disability.

*“A lot comes down to the compassion of the coaches … If they understand the situation then they will cater for it and give them the attention [they] need” [Parent #5]*

Participants also articulated that peer involvement and acceptance were strong motivators for children with disability to participate.

*‘He plays football (…) he’s never ever kicked a goal but nobody worries about that, … They always are really encouraging towards him” [Parent #6]*

Many participants described negative societal attitudes towards disability as a barrier to participation. Participants suggested some sports and recreation staff and physical education teachers lacked experience in disability and were fearful about including children with disability, or viewed it as a low priority.

*“Some teachers I’ve had in PE [physical education] they don’t want to like really listen ….[they say] we’re doing it this way and that’s it, you have to adapt to try and fit in” [Child #4, aged 15]*

Similarly, participants described how the parents of children with typical development could be openly negative about their child playing with a child with disability.

*“A few mums have said to me oh my son or my daughter’s picking up bad habits from your son” [Parent #7]*

Inclusive policies and support from sports governing bodies and all levels of government were considered to drive physical activity opportunities for people with disability and facilitate their inclusion. The role of schools in promoting physical activity was also discussed. Participants reported that some schools created opportunities for children with disability to be active and this facilitated their participation in physical activity in the wider community. This feature was ascribed more often to special schools than mainstream schools.

### One size does not fit all…it’s about choice

This theme was characterised by the idea that every child with disability was different, their particular needs were different and the type of activities that they and their family wanted to participate in were different.

Children and parents suggested the best way for providers of physical activities to find out how to include a child with disability was to ask them.

*“There’s no right or wrong about how you modify the rules, you ask the participants how they would find it easier for them (…) it’s no use assuming” [Sport and recreation staff #1]*

Parents stressed the importance of careful selection of activities with recognition that not all activities could be modified and some were inherently unsuitable. Meaningful activity was described as mainstream, structured, competitive sports for some children with disability, and for others, it was simple non-competitive, segregated or individual activities.

*“We played racquet ball, just me and [child] so he can chuck a tantrum, it’s not interfering with anybody, …” [Parent #8]*

*“I’ve been playing [footy] for 4 or 5 years. I found that everyone in my team is really supportive and nice and they treat me normally, they all give me a turn ….” [Child, aged 12 #5]*

Inclusive pathways with structured progression of participation were identified by sport and recreation industry personnel as being particularly important for children with disability. They described pathways as starting out in segregated classes, and progressing to individual activities, or social competitions and then moving on to mainstream or group activities or competitive sport. Often, activity opportunities were one-off programs and did not provide a pathway to become sufficiently competent so that children could progress to the next level.

*“All three boys could go [to soccer] and the two boys who were more able could be in the small-sided games and the other boy can start in the disability-specific one [but] there’s a clear pathway for him to go from that to the other activity with his brothers if he’s able and interested” [Sport and recreation staff #2]*

A lack of opportunities for children with disability was cited as a major barrier to their participation. Parents from regional and metropolitan areas reported marked variation in the availability of programs, and long waiting lists for segregated programs. Conversely, sport and recreation industry personnel discussed how programs were often not viable due to a lack of participants.

*“There’s lots of sport that kids can do in your local community but it’s not for special needs kids” [Parent #9]*

*“They need a certain number of people to make a program and the activity run or to make it financial” [Sport and recreation staff #3]*

Local activities, easily accessible by public transport, were cited as facilitators of participation.

*“Having opportunities reasonably local helps as well. I find [child] doesn’t like going on long car trips” [Parent #10]*

*Transport is such a battle for [families including a child with disability]” [Sport and recreation staff #4]*

### Communication and connections

Participants described a disconnect between families of children with disability, and the groups that promoted engagement in physical activity including schools, disability groups and the sport and recreation sector.

Physical activity programs for children with disability were reported to be poorly advertised. Parents talked about their difficulty in finding out about programs and how they relied on word of mouth and their own research to locate opportunities for their child.

*“We’ve heard of a number of organisations just by people telling us, other friends who have disabled kids informing us what groups are available” [Parent #11]*

Special schools were acknowledged to be a good source of information about available programs, unlike mainstream schools which were described as providing scant information. The problem with advertising was also raised by sport and recreation industry personnel who spoke about their difficultly in connecting with children with disability and their families.

*“We’ve tried a new approach through local Council (…), they’ve sent out the flyers for me because they know the names and the contacts but I can’t” [Sport and recreation staff #1]*

Participants suggested partnerships between physical activity providers, local councils, schools, disability groups and the health sector could better facilitate physical activity among children with disability. These partnerships could promote programs for children with disability, improve access to available opportunities, highlighted the importance of engagement in physical activity and help foster pathways between school and community sport. However, such partnerships were currently not widespread, particularly between the disability and sports sectors.

*“The disability sector has a very bad understanding of sport and recreation and how to get involved in that” [Sport and recreation staff #5]*

## Discussion

Our study adds to the available literature by exploring in more depth the facilitators of physical activity for children with disability, and by including the perspectives of sports and recreation industry personnel. The range and diversity of themes that emerged from the data illustrates the complexity of the issue, and is consistent with the conceptual model proposed by van der Ploeg [12] and with previous literature in both children [13] and adults [17] with disability.

Providing choice in physical activities children with disability can engage in was considered a key facilitator. Choice included segregated or integrated programs, type of physical activity, the level of participation (foundation skills to elite sports), individual or team sports, competitive or non-competitive activities, and the scheduling of programs. A complexity is that the needs of children with disability can change over time. There is also tension between the ideal scenario (providing meaningful choice) and the reality that programmes have limited resources to accommodate choice. Inclusive programs although more complex, might be more feasible, but may not be appropriate for every child with disability. However, there may not be a critical mass of children with disability living in an area to make a segregated program viable [24, 25]. Competitive sport is not for every child, whether they have a disability or not, but there are few non-competitive programs available. However, although previous literature suggested competitive team sports can exclude children with disability [26], our results suggest competition was seen as a positive. Sport and recreation industry personnel highlighted that many children with disability wanted to be involved in activity at a competitive level although it was often assumed they were only interested in ‘hit and giggle’.

Another facilitator of physical activity proffered by the participants was the concept of inclusive pathways. An inclusive pathway would provide a structured means of skills development. Having the requisite motor and social skills contributes to successful participation in physical activity among all children [25]. These skills are learnt through practice and early opportunities to develop them encourages participation by children especially when they experience success [25]. School is often where this practice happens. Unfortunately, children with disability do not always engage in physical education at school [27, 28]. Children with disability may also have fewer opportunities for mastering skills outside of school [29] because they are either excluded from community programs or their parents may not enrol them [26]. This means children with disability are potentially missing out on a range of opportunities to develop the skills they require to engage in physical activity. In addition, activity programs for children with disability are often short-lived [24, 26] such as ‘come and try’ days [25]. Through inclusive pathways children could achieve a sense of competence in an activity or skills before ‘graduating’ to the next level of difficulty. The ‘pathway’ would also provide a link at transition periods, for example, when moving from school-based activities to community-based activities.

Inclusive pathways may also facilitate participation through better development of connections with stakeholders. A disconnect between stakeholders from the disability, sport and recreation, education, local government and health sectors, was identified as a key barrier to engaging children with disability in physical activity. The perception of participants was that stakeholders operated independently without collaborating with each other and that no sector saw it as their responsibility to help engage children with disability in physical activity. This concept has not been explored much within the literature; one study including adults with disability reported a lack of collaboration between organisations as a barrier to physical activity [16] and one study suggested strong partnerships between relevant organisations as a facilitator of activity for children with disability [26]. Efforts to bring together stakeholders should be encouraged as it would help maximise expertise on disability issues, and could facilitate better activity opportunities for children with disability through the development of pathways.

Parents and families are crucial to whether a child with disability is physically active. Parents are a child’s primary advocate and support their participation financially and practically. The value parents place on physical activity is indicative of the level of their child’s participation [30] and parental and child beliefs about physical activity are strongly related [29]. A majority of parents understand the benefits of physical activity, and are happy for their child to be active [31]. Their main issue is how to make it happen so they can balance the needs of family members [32] and identify suitable programmes for their child [33, 34]. Better marketing of physical activity opportunities for children with disability was one strategy parents felt could facilitate participation as most parents reported that word of mouth was their primary or only source of information [31]. Marketing could encourage participation by including information on program goals, skill levels, instruction, staffing, and transport [25]. It also needs to be inviting, particularly for first time users, and distributed locally where the target group will find it [25, 31].

Participants indicated that social barriers to participation (such as the attitudes of parents, staff and peers) were more influential than other types of barriers. Negative attitudes, societal stereotypes of disability and a lack of acceptance by peers are well documented barriers to participation [26, 35] as they inhibit interest in physical activity among children with disability [33]. Changing attitudes is difficult, but contact theory suggests that the experience, of getting to know or working with someone with a disability, can positively change attitudes [36]. Disability awareness programmes for staff and peers may help to minimise misunderstandings about the needs and abilities of people with disability [25, 29]. They might be helpful in developing knowledge and skills about how to adapt activities [26, 27, 29, 37] and encourage peer interaction to create a welcoming environment. Positive interactions between children with and without disability are not automatic [26] and require planning but they offer opportunities for friendships for children with disability [31], and awareness of disability for children with typical development [38].

The strength of this study is that it is an in-depth study of the barriers and facilitators to physical activity for children with disability and one of the first to include the perspective of sport and recreation personnel. Previous studies [17] have described the barriers and facilitators to participation in physical activity for adults with disability only or have provided a preliminary overview of the perspectives of sports and recreation industry personnel [15]. Collating these perspectives with those of parents and children with disability helped to triangulate the data and provide deeper understanding. A limitation was that participants self-selected into the study; those who were more physically active may have been more inclined to be involved. However, if this was the case, these participants would have been potentially more able to provide insight into what facilitated children with disability to be active and resourceful in overcoming existing barriers to activity.

## Conclusion

As the long-term consequences of physical inactivity can lead to serious secondary health problems among people with disability, understanding the factors that influence participation in physical activity is important to help design successful interventions and strategies that increase their level of engagement in activity from an early age. Our results confirm children with disabilities’ need for the early attainment of motor and social skills, the integral role of families and their need for support, and that societal attitudes continue to influence children with disabilities’ participation. Other themes that emerged from the data were the need for inclusive pathways that encourage ongoing participation as children grow or as their skills develop and for the development of better partnerships between key stakeholders from the disability, sport, education and government sectors. Based on these themes, possible individual, social and policy level strategies for improving participation in physical activity among children with disability which require further investigation are presented in Table 3. The broader understanding of the barriers and facilitators to physical activity for children with disability gained through this study is essential for the design and implementation of effective interventions, strategies and policies to promotion their participation. We expect our findings will be useful to health professionals, health promotion agencies and the sport and recreation sector to help increase the amount of physical activity that these children perform.

**Table 3:** Possible strategies to improve participation in physical activity among children with disability

*Individual level strategies*	
1. Incorporate practical based instructor training in disability 2. Ask children with disability and their families their preferred activity choices 3. Introduce flexible or subsidised payment options for families of children with disability 4. Encourage children with disability to participate in physical activity from early childhood	
*Social level strategies*	
5. Lessen the burden on parents of children with disability through financial or social support or incentives 6. Introduce flexible funding arrangements for sports organisations 7. Promote physical activity programs that children with disability can participate in 8. Ensure children with disability meaningfully participate in physical education at school	
*Policy level strategies*	
9. Develop partnerships between the sport and disability sectors, local government, and schools 10. Encourage positive societal attitudes to disability	

## Additional file

Additional file 1:
**Focus group schedule - parent group.** (DOC 34 kb)
