# Supplementary material for: Perceived barriers and facilitators to participation in physical activity for children with disability: a qualitative study
Source: BMC Pediatr. 2016 Jan 19;16:9. doi: 10.1186/s12887-016-0544-7 (PMC4717582; doi:10.1186/s12887-016-0544-7)
Supplement: Additional file 1: — Focus group schedule - parent group. (DOC 34 kb) [file 12887_2016_544_MOESM1_ESM.doc]

**FOCUS GROUP SCHEDULE- PARENT GROUP**

1. Introduction to the focus group moderator and assistant moderator
2. Explain the purpose of the focus group is to discuss the facilitators (supports, enablers or things that help) and barriers (challenges or hindrances or things that stop or prevent participation) to participation in physical activity for children with disability.

By the term physical activity we mean any structured or unstructured activity where your child is physically active, such as walking the dog, going swimming, active play, doing exercise, or playing sports or physically active games at school or in a community group.

**QUESTIONS**

1. Ask the group to go round the table and introduce themselves, tell us briefly about your child and what physical activities they currently take part in
2. What are some of the key things that have helped or facilitated your child to get involved in physical activity?

(Potential prompts)

- *Tell us about your role as parents in supporting your child to take part in physical activity*
- *Have you found integrated or segregated physical activities to be more beneficial for your child?*
- *Have social attitudes towards children with disabilities influenced how active your child is?*

1. What have been some of the challenges or barriers to your child participating in physical activity?

(Potential prompts)

- *Tell us about your experience in accessing your local sporting or physical activity facilities*
- *What influence does cost or money have on how active your child is?*
- *What influence does transport have on how active your child is?*

1. Ask the group to put their creative hats on and tell us what supports would help your child to participate in physical activity to overcome some of these barriers?
2. What key recommendations would you make for including children with disabilities in physical activity?
